# What Influences Women to Adhere to Pelvic Floor Exercises after Physiotherapy Treatment? A Qualitative Study for Individualized Pelvic Health Care

**DOI:** 10.3390/jpm11121368

**Published:** 2021-12-14

**Authors:** Beatriz Navarro-Brazález, Fernando Vergara-Pérez, Virginia Prieto-Gómez, Beatriz Sánchez-Sánchez, María José Yuste-Sánchez, María Torres-Lacomba

**Affiliations:** Physiotherapy in Women’s Health (FPSM) Research Group, Physiotherapy Department, Faculty of Medicine and Health Sciences, University of Alcalá, 28871 Alcalá de Henares, Spain; b.navarro@uah.es (B.N.-B.); fernando.vergara@uah.es (F.V.-P.); beatriz.sanchez@uah.es (B.S.-S.); marijo.yuste@uah.es (M.J.Y.-S.); maria.torres@uah.es (M.T.-L.)

**Keywords:** pelvic floor muscle exercises, pelvic floor dysfunction, qualitative research, therapeutic exercise, therapeutic adherence, women’s health physiotherapy

## Abstract

Conservative treatment of pelvic floor dysfunction (PFD) includes therapeutic exercise for pelvic floor muscle (PFM) training or other complementary exercise modalities, such as hypopressive exercises. However, the long-term effectiveness of the conservative treatment depends on a patient’s adherence to the exercises and the integration of professional health advice into their daily life. The objective of this study was to establish the adherence experience of women with diagnosed PFD in home-based exercises after an intensive face-to-face physiotherapy treatment. A qualitative study from an interpretive paradigm was developed. Semi-structured individual and group interviews were performed 6 months after finishing individual physiotherapy treatment. The interviews were recorded, fully transcribed and analyzed thematically by creating categories. Thirty-one women were interviewed. The women reported that their adherence to home PFM exercises depended on the exercise program itself, its efficacy, their personal experiences with the exercises, intrinsic factors such as self-awareness or beliefs, and extrinsic factors, such as professional or instrumental feedback. Thus, therapeutic adherence could be more likely with effective physiotherapy programs that include mutually agreed home exercises and simple movements women can build into their daily lives. Improving awareness and knowledge of the pelvic region and the importance of PFM treatment as well as consideration for potential worsening of PFD will also encourage women to adhere to the exercises.

## 1. Introduction

The pelvic floor muscles (PFM) play an important role in the preservation of urinary and anal continence, in pelvic organ support and in sexual function, among others [1]. The weakness and loss of PFM properties are associated with the development and maintenance of pelvic floor dysfunctions (PFD) [2,3]. Prevalence studies estimate that PFD affect up to 40% of women [4], including symptoms of urinary incontinence (UI), pelvic organ prolapse (POP), and anal incontinence (AI), which are the most frequent PFD. The first line of treatment for mild PFD is focused on specific PFM exercises [5,6], which can involve a therapeutic education program [7] to provide knowledge and self-management strategies to women. In addition, global exercise modalities, such as hypopressive exercises, have recently begun to be included in PFD treatment [8,9,10], to train the PFM in coordination with posture and adjacent muscles. However, the long-term effectiveness of conservative treatment does not seem to depend exclusively on the exercise method. Patient adherence to a prescribed exercise program and to health professional advice seems to be one of the determining variables to ensure the success of the treatment in the short and long term [11].

The determining factors of therapeutic adherence to PFM exercises have been analyzed and classified based on patient personal parameters or factors dependent on physiotherapeutic performance. Motivation, perceived self-efficacy and benefit expectations are identified as personal facilitating factors, while misconceptions, false beliefs or low body awareness are presented as personal adherence barriers. Regarding physiotherapy treatment, a structured program and close supervision are positive adherence factors, and a high number of sessions and abstract concepts are associated with poor adherence [12,13,14].

Hay-Smith et al. [15] inquired about the experiences of women in performing PFM exercises at home and observed that a high degree of self-efficacy was needed, which could be improved with individualized educational programs. Recent qualitative studies in women with PFD point to the patient’s demand for [16] and the perceived usefulness [17] of a structured educational program on the functions and dysfunctions of the pelvic floor, on risk factors and on coping strategies. However, further studies to understand and delve into the patient’s experiences are required to develop more long-term effective and patient-centered programs [18,19].

In a previously published randomized clinical trial [8], women who experienced stress or mixed urinary incontinence, anal incontinence, or mild pelvic organ prolapse received physiotherapy treatment. Participants were randomly allocated to either PFM training and an educational strategy (PFMT group), or to hypopressive exercises and an educational strategy (HE group), or to PFM training plus hypopressive exercises and an educational strategy (PFMT+HE group). The three programs were conducted individually and face-to-face by the same physiotherapist specialized in women’s health. The physiotherapy treatment lasted 8 weeks, with two visits of 45 min each week. Moreover, women were advised to carry out 15 min of daily home exercises based on the specifications of the physiotherapist depending on the intervention group. Despite the difference between the study exercises, the three groups showed similar adherence data, not being able to understand or deepen the reasons for continuing or abandoning the home guidelines.

Therefore, the aim of the present study was to establish the experience of women to adhere to home exercises recommended by their physiotherapist after completing an intensive face-to-face treatment, which included PFM exercises and/or hypopressive exercises and an individually tailored educational program.

## 2. Materials and Methods

### 2.1. Research Team and Reflexibility

From October 2014 to June 2016, semi-structured individual interviews and focus groups were conducted. The personal interviews were carried out by the physiotherapist who performed the 2 months of individual treatments. This assumed that the participants knew the physiotherapist, and a prior relationship of trust could have been established. The physiotherapist (B.N.-B.) was a woman with more than 5 years of professional experience in women’s health and with previous experience in qualitative interviews. The focus groups were led by another woman physiotherapist (B.S.-S.), a university professor, who also specialized in women’s health and was experienced in qualitative research interviews. She was not connected with the study in order to achieve the appearance of a new perspective [20], and the participants did not know her. All the interviews began with the presentation of the objective of the study, the guarantee of the confidentiality of the contributions and the request for permission to record the audio of the interviews.

### 2.2. Study Design

A qualitative descriptive study was developed from an interpretative paradigm design to present the women’s perspectives on the phenomenon of adherence [21] to the pelvic floor exercise program after a randomized clinical trial [8]. The Clinical Research Committee of the Principe de Asturias Hospital (OE20/2013) approved the study, which was registered at ClinicalTrials.gov (NCT02259712). The consolidated criteria for reporting qualitative research (COREQ) were followed for reporting [22].

### 2.3. Participant Selection and Setting

Purposive sampling was used to select women from the three intervention groups. Women who were included in the randomized clinical trial and were in the follow-up period after the physiotherapy treatment were invited to participate in the qualitative study. The inclusion criteria were to have completed the 2 months of physiotherapy treatment in any of the three study groups, for a period of between 3 and 6 months to have elapsed after its completion, to have availability to attend the interview, speak Spanish fluently and to freely agree to participate. Since no statistically significant differences were found in the adherence of the three study groups, the qualitative study was proposed and aimed at all participants, regardless of the assigned treatment group. The capture of the individual interviews was carried out in person during the physiotherapy reviews, inviting participants to carry out the interview at a time that best suited them. Group interviews were scheduled, and the participants were contacted by phone to give them a face-to-face appointment.

A total of 20 individual semi-structured face-to-face interviews were completed: nine interviews of women allocated to the PFMT group, six interviews of women in the HE group, and five interviews of participants who were in the PFMT+HE group. Furthermore, three group interviews were conducted, one for each intervention group with the participation of three to five women in each group. Three participants rejected the interview due to time incompatibility. The appointments were developed in a private room at the FPSM-RG laboratory at the University of Alcalá (Madrid, Spain). During the interviews, only the participants and the interviewer were present.

Since the age of the participants [23], the PFD symptoms, or the improvement found with the physiotherapy treatment could influence the results of the interviews [14], a descriptive analysis of the sample was carried out. This description only provides a descriptive character of the participant and not a criterion for interpretive analysis. To verify normal distribution of the data, the Kolmogorov–Smirnov statistical test was used. The normal quantitative variables were described with means and standard deviations. The categorical variables were described with absolute frequencies and percentages. For this statistical analysis, the IMB SPSS Statistics version 20 software was used.

The mean age of the participants was 49.74 (10.78) years, with a BMI of 26.01 (4.88) kg/m^2^, 16 (51.6%) of them had experienced menopause, and in relation to the history of pregnancy and childbirth had an average of 2 (1) vaginal deliveries. Annual household income indicated that the study population belonged to the middle class; 35.5% of the women had a university education, 51.6% had a basic education, and one participant indicated that she had never attended school. All the participants suffered from some PFD, since it was an inclusion criterion, presenting UI in 87.1%, IA in 48.4%, and POP in 51.6% of the women. Comparison of PFM strength and quality of life survey results before and after the physiotherapy treatment indicated improvements in all participants. Table 1 shows the characteristics of the sample in more detail.

### 2.4. Data Collection

Prior to the start of the interviews, a group of three physiotherapists, specializing in women’s health (M.T-L. and M.Y-S.) and qualitative research (F.V.-P.), and a gynecologist developed referral questions (Table 2). After the guide questions were prepared, they were presented to the interviewers to consider if they needed any changes. A pilot test was carried out with a participant. This interview was excluded because it was not recorded and was only conducted to ensure the understandability and relevance of the questions.

All the interviews included in this study were audio recorded with participant permission. No field notes were taken, the interviews were conducted only once, and the interview transcriptions were not returned to participants.

The mean duration was 24 min for individual interviews and 65 min for focal group interviews. Data saturation was considered when the information collected was consistent with the previous interviews. This fact was shared and discussed with the physiotherapists who participated in transcribing the interviews.

### 2.5. Data Analysis

Five physiotherapist members of the FPSM-RG (B.N.-B., F.V.-P, V.P.-G., M.T.-L. and B.S.-S.) manually transcribed the recorded interviews. Different codes were established as part of the intervention group allocation, to sort the interview (individual or in group) and the participant intervention order, with the purpose of securing the women’s anonymity. Researchers’ triangulation process was performed, where three physiotherapist members analyzed the resultant texts (B.N.-B., F.V.P and M.T.-L.). In an iterative consensus process, the researchers conducted an open and axial coding since a previous theoretical framework was not used, as well as related codes and categories. The transcription encoding was performed using the ATLAS.ti version 6.1 software (Scientific Software Development GMBH, Berlin, Germany). The interviews and the categorization of the themes were carried out in Spanish. Quotes and categories were later translated into English.

## 3. Results

Five themes emerged from the literal transcription analyses: the exercise program, the program efficacy, personal experiences with exercises, intrinsic factors, and extrinsic factors (Table 3).

### 3.1. The Exercise Program

Inclusion in the program itself was identified as a motivating factor. Many women expressed the need for several appointments with health professionals about their PFD to be informed about a physiotherapy treatment. Moreover, the program was free, costly at a private level and almost non-existent in public services.

HEG4: “The only thing they told me about was a possible operation”.

PFMTG3: “Nor the gynecologist, nor the midwifery; no one tell you anything, there is no information”.

On a social level, PFD did not seem to be recognized either, as the following participant pointed out:

PFMTI3: “It is a pity that I am not so heard, as for example if you have a bone problem you go to trauma, but I do not know about you if it is not for the last doctor.”

The moment when women should access the program was also mentioned because many considered that conservative treatments should be implied before the symptoms showed up or when they were minimally aware.

PFMT+HEG4: “As a birth preparation is stablished, it should be a repaired post-labor treatment”.

HEG1: “and in menopause...”

Furthermore, participants were generally satisfied with the exercise program because of the pleasant relationship with the physiotherapist, the knowledge gained, and the physical and mental well-being acquired.

PFMTI1: “It was worth it, I am delighted, and I would repeat it, for me it has been a very good and very positive experience.”

### 3.2. Program Efficacy

Close to the program satisfaction were the positive results achieved with the physiotherapy treatment. The improvement in perceived symptoms was identified as a key factor in treatment adherence:

HEG1: “Since I perform the exercises, I feel better, more secure. Before that, I avoid myself to do things, because it was uncomfortable… But not anymore! I feel safer”.

Many women showed signs of general well-being, indicating that they felt happy, good about themselves, with better self-esteem and with the ability to return to a normal life, in which they could control their symptoms. That feeling of well-being helped them continue with the treatment:

PFMTI1: “I keep my self-esteem high, my relationships normal and my life normal, everything positive that entails, I have achieved everything I wanted, I am interested in keeping it.”

These benefits provided by the program were also specified in the physical symptoms of the pelvic floor. In the first place, the participants’ previous experience with their physical symptoms played a significant role, since each of these symptoms diminished an aspect of their daily life:

PFMT+HEI5: “Every two steps I was afraid of cough, okay, not much, droplets, but today one, later another, another, another...”

Additionally, the fact that, after the exercise program, they had managed to minimize these symptoms, was also an adherent factor:

PFMT+HEI3: “Before I was very overwhelmed, it was a small cough and now, I cough calmly so to speak.”

However, in some participants in which the symptoms were not totally cured, the limited efficacy of the treatment was a barrier to continuation at home.

PFMTI5: “I don’t have a 100% of improvement. […] Doing big sacrifices, but at the end, I know that always some drops will escape, so…”.

### 3.3. Personal Experiences with Exercises

Participants revealed that they required a quiet environment and concentration to perform the exercises correctly, which was impracticable due to their family and work duties.

PFMTI4: “I would need more order in my life […] I have three kids, the work, the housework, every day, I’m always nervous”.

Moreover, women expressed other health issues that made it difficult to continue the exercises, such as colds, allergies, low-back pain, or vaginal infections. Integrating the PFM exercises with other daily activities was essential, and this was closely connected with the perceived difficulty of the exercises. Exercises were said to be performed while walking, driving, doing the housework, or going to the gym. Furthermore, the personal preferences for the exercises (the type of exercise, posture, the demanded conditions of the exercise) and the perceived efficacy of one over another, influenced the adherence.

PFMTI4: “I only practice the maintenance exercise, because I think that to gain force it’s ok”.

### 3.4. Intrinsic Factors

The self-perceived symptoms, the self-perceived treatment efficacy, and the ability to reproduce exercises efficiently as well as to correct themselves were important in adherence. Moreover, the participants’ beliefs, their immediate environment and the normalization of symptoms of urinary leakage in society affected adherence.

PFMTG3: “Some of my friends do hypopressive exercises, and I’d like to be in the hypopressive group, because I think that they’re really good”.

The newly acquired awareness of responsibility changed the women from passive agents to active agents in the improvement of their condition.

HEI3: “Now the responsibility is mine, if I don’t do anything… there is no magic”.

### 3.5. Extrinsic Factors

Regular contact with the PT, the use of biofeedback devices and mirrors, and the posterior assessments were named as motivating factors.

PFMTI8: “The gynecology gave me a paper with some exercises. But I didn’t understand them, nor fancy doing them. When I came here, I saw the importance they have”.

Nevertheless, finishing the continuous physiotherapy treatment required some women to withdraw from the weekly PFM training. The evaluation and feedback provided by other health professionals also was a condition of adherence to the exercises.

## 4. Discussion

This study shows the adherence of women with PFD to the exercises recommended by their physiotherapist 3 to 6 months after an intensive treatment. To our knowledge, this is the first qualitative research that interviewed women with different mild forms of PFD, who underwent an individualized treatment based on PFM guided exercises with biofeedback and/or hypopressive exercises. Five categories emerged from the thematic analysis that explained the adherence-modifying factors: the exercise program, the program efficacy, the personal experience with exercises, patient’s intrinsic aspects and extrinsic factors. Each theme contained related codes, from which positive and negative aspects for adherence were extracted.

Other qualitative studies emerged from randomized clinical trials. Our study is consistent with the study by Hyland et al. [24]. They presented the interviews of five women with POP between 5 and 24 months after participating in prescribed PFM exercises and selected positive adherence codes related to the ease of performing the exercises anywhere and their association with everyday moments. Family duties were emphasized as a determining aspect of adherence and maintenance of PFM exercise over time, a complicated challenge influenced by the low self-efficacy perception. The perception of self-efficacy has been identified as one of the main predictors of adherence to the maintenance of home PFM exercises [25]. To reinforce self-efficacy, the use of motivational interviewing has been proposed [15,26] and agreement reached on individualized goals based on the motivational stage of the patient [27], aspects that were included in the three intervention groups.

Adherence rates in our three study groups were similar. In the PFMT group, 71.9% continued exercising weekly at home, 61.3% in the HE group, and 67.7% in the PFMT+HE group [8]. Hay-Smith et al. [16] focused on PFM exercise comprehension and found that participants had trouble distinguishing the different exercise interventions, which could alter the outcome of their study. In our study, we did not identify confusion by the participants in identifying the different exercises, probably because the PFM exercises and the hypopressive exercises are different in their execution [8]. Our qualitative research showed that women found it difficult to continue a daily home exercise routine, especially if they perceived the exercise to be difficult, exhausting, or incompatible with a simultaneous activity; thus, we were surprised by the high adherence data found in relation to hypopressive exercises. In Spain, hypopressive exercises were popular among women; in fact, a participant from the PFM group revealed in her interview that she would have liked to join the HE group. This social support for an exercise modality could be a significant factor of adherence [28]. Furthermore, the treatment effectiveness perceived by the participants appeared as a positive factor in maintaining its practice at home [29], and in the three intervention groups, the women achieved clinically relevant improvements in their quality of life, which could also explain the similar adherence results.

Women valued the need for a treatment that produced improvements in relation to the symptoms of the pelvic floor, provided them with a feeling of well-being and generated secondary improvements, such as the minimization of low back pain. These findings are in line with the promotion adherence results found by Alewejnse et al. [7], who showed that a well-designed physiotherapy protocol may downplay the addition of a health education program. However, the lack of information and false beliefs have been identified in women with UI [16], assuming educational programs are an essential tool for the health education of these women. In fact, the participants in our study highlighted the knowledge acquired as a positive factor of adherence, finding in other studies how women perceive that it was a useful learning experience for life. The feedback provided by the physiotherapist and the periodic evaluation visits also appeared as facilitating aspects of adherence [30].

Several barriers to adherence were identified based on the experiences of the women in our study. They included difficulty in accessing a pelvic floor physiotherapy service in relation to ignorance of its existence and its high price, as it is not generally integrated into public health services [29,30,31]; whether treatment is started with advanced symptoms; and failure to achieve complete resolution of the symptoms. Regarding the time when the physiotherapy program should start, the women agreed that around postpartum/first delivery could be a good time. Despite the opinions of women, qualitative studies show that postpartum women are unaware of PFD that may appear after childbirth, and that they believe it is an inevitable consequence and do not know about the treatment alternatives available [31,32]. Grant et al. [33] interviewed 31 women who had given birth in the last 5 years and found that women needed more information and support for proper performance of the MSP exercises, which could also be facilitated by the use of new technologies, such as the creation of an app. Other identified barriers to adherence were related to beliefs about normalization or taboo subjects [31,33], a lack of self-care [34], prioritizing family needs [24], or job obligations [30].

Pakbaz et al. [34] identified feeling ignored by healthcare professionals as an obstacle of attention demand; an assumption in our study was the feeling of gratitude for care as a positive factor for adherence. This appreciation could enable the patients to have good experiences since the physiotherapist who guided the treatment and prescribed the exercises was also the interviewer, which was a possible limitation of the study. To minimize this fact, group interviews were performed by an external physiotherapist who also specialized in women’s health. However, to enlist the same intervening physiotherapist for interviews was an advantage because she provided confidence to the interviewed participants, and her knowledge about women was used to interpret the experiences and categorize the information [24]. A strength of our study is that the questions were agreed upon by different health professionals and the process of triangulation and analysis of the information was carried out by five physiotherapist experts in women’s health and qualitative research.

The practical implications derived from the present study can be directed first to the planning of effective physiotherapy programs that promote the empowerment of women, and second to the evaluation and management of contextual factors that may be positive or negative. Knowing and exploring both the beneficial factors and the adherence barriers present in each woman would allow the physiotherapist and patient to agree on realistic home exercises [30] and in meaningful environments. For future research, it is considered essential to explore and deepen the experience of women regarding their needs related to improvement in their PFD, their opinions on current management and the possibilities for them to take an active role in their own recovery. Designing mixed-method studies or qualitative studies after intervention research could delve into the personal reasons for adhering or not to a treatment plan, especially important in chronic or long-terms disorders such as PFD.

## 5. Conclusions

In the present qualitative study on the experience of maintaining home exercises after intensive physiotherapy treatment in women with PFD, five central themes were identified: the exercise program, program efficacy, personal experience with the exercises, intrinsic factors, and extrinsic factors. Interventions with perceived effectiveness and easy exercises suitable for integration into daily life would enhance therapeutic adherence. Providing knowledge so that women recognize their pelvic floor and the importance of PFM exercises as well as take an active role in their self-care process should be a goal of health professionals, also reinforced by professionals.

## Figures and Tables

**Table 1:** Demographic and clinical data of the participating women.

Clinical Characteristics	*n* = 31	
Age years, (SD)	49.74 (10.78)	
BMI kg/m^2^, (SD)	26.01 (4.88)	
Menopause, *n* (%)	16 (51.6%)	
Parity, (SD)	2 (1)	
Education		
Never went to school *n* (%)	1 (3.2%)	
Primary school *n* (%)	7 (22.6%)	
Secondary school *n* (%)	9 (29%)	
Vocational Education and Training/ Certificate of Higher Education *n* (%)	3 (9.7%)	
University degree *n* (%)	11 (35.5%)	
Annual income		
<12,000 €	8 (25.8%)	
12,000−24,000 €	9 (29%)	
24,000−36,000 €	11 (35.5%)	
36,000–48,000 €	2 (6.5%)	
>48,000 €	1 (3.2%)	
Pelvic floor dysfunction	31 (100%)	
UI, *n* (%)	27 (87.1%)	
AI, *n* (%)	15 (48.4%)	
POP, *n* (%)	16 (51.6%)	
Modified Oxford scale	Pre-treatment	Post-treatment
0 *n* (%)	1 (3.2%)	0
1 *n* (%)	3 (9.7%)	1 (3.2%)
2 *n* (%)	4 (12.9%)	1 (3.2%)
3 *n* (%)	15 (48.4%)	3 (9.7%)
4 *n* (%)	8 (25.8%)	7 (22.6%)
5 *n* (%)	0	19 (61.3%)
Manometry cmH2O, (SD)	20.23 (14.71)	30.04 (16.94)
PFDI-20 total, (SD)	76.44 (43.11)	49.23 (43.8)
PFIQ-7 total, (SD)	23.81 (52.37)	9.52 (23.81)

*n*: Number; SD: Standard deviation; BMI: Body mass index; UI: Urinary incontinence; AI: Anal incontinence; POP: Pelvic organ prolapse; PFDI-20: Pelvic Floor Distress Inventory Short Form; PFIQ-7: Pelvic Floor Impact Questionnaire Short Form.

**Table 2:** Guide questions for the individual and focal group interviews.

Number	Question
1	What do you think is the effect of the exercises? What do you think they are good for?
2	At what time of the day do you practice them?
3	What exercises do you practice the most? Why?
4	What exercises do you practice the least? Why?
5	What do you think makes it easier to practice the exercises?
6	What do you think makes it difficult to practice the exercises?
7	What responsibility do you think you have to improve your symptoms?
8	Are the exercises worth doing?
9	Was attending the pelvic floor physiotherapy program worth it?
10	Have you included exercises at some point in your daily life? When?
11	Do you associate the exercises with any situation with a preventive objective?

**Table 3:** Main themes and codes extracted from participants’ interviews.

Themes	Code	Positive	Negative
Exercise program	How to access	- The privilege to be attended to. - This program was free, and in private health care would have a high cost.	- No derivation for other health professionals. - Low public awareness of program existence. - Not all women can benefit from this program.
	Access time	- Started when it could be adapted to own schedule. - Started with mild symptoms.	- Lack of preventive physiotherapy treatment. - Started when symptoms were difficult to resolve with conservative treatment.
	Program satisfaction	- General personal satisfaction. - Nice attention from physiotherapist. - The feeling of being listened to and attended to.	
Program efficacy	Pelvic floor physical symptoms	-Lower leakage. - Lower heaviness sensation. - Not feeling a vaginal bulge. - Improvement of pelvic-perineal pain. - Sexual improvement. - Control of urinary urgency.	- Not a complete cure.
	Secondary physical symptoms	- Improved lower back pain. - Abdominal reduction. - Abdominal muscle strength.	- Back pain when performing exercises.
	Well-being	- Improved self-esteem. - Feeling of well-being.	
	Functional improvement	- Returning to sports routines. - Not fearing to cough, laugh, sneeze or lift weight.	
	Knowledge	- Self-control of symptoms. - Self- control of risk factors. - Awareness of treatment importance. - Being capable of taking responsibility for own dysfunction. - What to do when symptoms worsen. - Who to turn to when symptoms worsen.	
Personal experience with exercises	Conditions		- Family and housework duties. - Job duties. -The need for quiet environment. - Co-existing health problems.
	Dedication	- Adopt a daily or weekly routine. - Only a few minutes a day.	- Too much physical effort. - Too much mental effort. - The need for time to do exercises.
	Integration	- Automatize PFM contraction. - Perform anywhere. - Perform together with other activities.	- The need for practice. - To know when and where to integrate exercises.
	Exercises type	- Personal preferences. - Feeling of exercise efficacy. - Feeling of doing it well. - Helping to relax.	- Exhausted physical effort. - Difficult postures. - Not feeling PFM contraction. - Difficult to integrate. - The need for concentration. - The demanding conditions for exercise.
Intrinsic factors	Self-awareness	-Helping to understand what is happen in own body. - Self-care. - Positive biofeedback. - Improved self-confidence.	
	Strategies	- Developing own moves.	
	Beliefs	- Confident of treatment efficacy. - Fear of getting worse.	- To normalize the dysfunction. - Not openly talking about PFM problems. - Believing other treatments are better.
	Responsibility	- Commitment to themselves. - Commitment to physical therapists.	- Forgetting exercises. - Disinclined. - Prioritizing the problems of their family members- Not take care of self.
Extrinsic factors	Professional feedback	- Regular contact with the physical therapist. - Learning how to perform exercises correctly. - The possibility of solving questions. - Motivation. - Professional reinforcement.	- The need to visit the health center. - Dependence on health professional.
	Instrumental feedback	- Own reward. - Correcting the exercises. - Seeing progress. - Helping to remind about exercises.	

## Data Availability

More information on the data presented in this study can be requested from the corresponding author.
